# Social determinants of antidepressant continuation during pregnancy in the USA: findings from the ABCD cohort study

**DOI:** 10.1007/s00737-024-01470-0

**Published:** 2024-05-14

**Authors:** Marc Dupuis, Kristie Rebecca Weir, Renata Vidonscky Lüthold, Alice Panchaud, Stéphanie Baggio

**Affiliations:** 1 Institute of Primary Health Care (BIHAM), University of Bern, Mittelstrasse 43, Bern, 3012 Switzerland; 2 Graduate School for Health Sciences (GHS), University of Bern, Bern, Switzerland; 3 Sydney School of Public Health, Faculty of Medicine and Health, The University of Sydney, Sydney, Australia; 4 Service of Pharmacy, Lausanne University Hospital and University of Lausanne, Lausanne, Switzerland; 5 Population Health Laboratory (#PopHealthLab), University of Fribourg, Fribourg, Switzerland

**Keywords:** Antidepressants, Continuation, Discontinuation, Pregnancy, Social determinants

## Abstract

**Purpose:**

Patients and healthcare professionals overestimate the risks of using antidepressants during pregnancy. According to current literature, approximately half of people stop taking an anti-depressant medication when they become pregnant. Discontinuing antidepressants during pregnancy increases risks of postnatal relapses. Factors like socioeconomic status, education, and planned pregnancies play a role in the decision to continue antidepressant medication, which can worsen disparities in maternal and child health. Our aim was to identify the sociodemographic factors associated with antidepressant continuation after awareness of pregnancy.

**Methods:**

We used representative data from the Adolescent Brain Cognitive Development (ABCD) study that captures maternal medication during pregnancy. We identified women who used antidepressants before awareness of their pregnancy. We calculated crude and adjusted associations between sociodemographic factors and continuation of antidepressant medication during pregnancy. Our model included age, education, ethnicity, first language, household income, living with a partner, having planned the pregnancy, pregnancy duration and smoking during pregnancy.

**Results:**

In total, 199 women continued antidepressants and 100 discontinued. The logistic regressions resulted in only one significant factor: first language. Native English speakers were more likely to continue medication than other mothers (adjusted *OR* = 14.94, 95% CI = [2.40; 291.45], *p* = .015).

**Conclusions:**

Language differences were associated with continuation of antidepressants. Non-native English speakers were more likely to discontinue antidepressants, which may lead to health inequities. This finding should be taken into account to reinforce information about the limited risks of antidepressants among people with non-English speaking backgrounds in the USA.

## Introduction

Affective disorders such as depression are the most common mental disorders experienced during pregnancy. Depression is experienced in about 10% of pregnancies (Falah-Hassani et al. 2017; Moore Simas et al. 2023) and antidepressants are commonly used in pregnant women. Pregnant women and health care professionals largely overestimate the risks associated with antidepressant use during pregnancy (Lupattelli et al. 2014; Nordeng et al. 2010; Petersen et al. 2015). Side effects such as nausea, digestive issues, insomnia, and drowsiness are frequently reported during the first weeks of treatment, which can also demotivate pregnant patients who already experience such symptoms. Consequently, more than 50% of women discontinue antidepressant treatment during pregnancy (Cabaillot et al. 2021; Liu et al. 2022; Lupattelli et al. 2023; Trinh et al. 2023a), which can lead to negative consequences for themselves and their child such as relapses (Cohen et al. 2006) and conduct disorders among children.

Prior research has signaled that sociodemographic factors influence pregnancy outcomes. Though scarce, recent research suggests that social determinants may influence antidepressant continuation during pregnancy. A previous study showed that younger maternal age and a lower level of education increase the probability of discontinuing antidepressants (Liu et al. 2022). Moreover, social determinants of antidepressant continuation had been identified among a general population of patients (Olfson et al. 2006). Further studies are needed to explore other social determinants and to identify potential health inequalities.

we aimed to identify which social determinants are important for continuation of antidepressants among women when they become aware of their pregnancy. We investigated the associations between sociodemographic factors and continuation of antidepressant medication during pregnancy using data from the Adolescent Brain Cognitive Development (ABCD) study (Garavan et al. 2018; Jernigan et al. 2018).

## Methods

### Study design and setting

In the present study, we used data from the two first waves of ABCD, a large cohort study on adolescents that includes data from their parents on prenatal exposure to medications and illicit drug use before and after becoming aware of pregnancy. ABCD is an ongoing nationwide prospective study conducted by 21 universities and hospitals in 16 states from the USA: California, Colorado, Connecticut, Florida, Maryland, Minnesota, New York, Oklahoma, Oregon, Pennsylvania, South Carolina, Utah, Vermont, Virginia, Washington, and Wisconsin (Garavan et al. 2018). Though ABCD mainly consists of brain imaging sessions among preadolescents and adolescents, it also includes in-person interviews with parents, including *biological mothers* who were the participants of interest for our study. Parents were involved in ABCD to provide information about themselves including their drug use during pregnancy ten years before recruitment, current health status and the early life course of their children.

Families were sampled using a multi-stage probability sampling strategy to maximize representativeness of the subpopulations and were recruited through elementary public and private schools to cover almost the entire preadolescent population of 9 to 10 year old. The baseline investigation took place from 2016 to 2018 when children were aged about 10 years and the first follow-up took place one year later. Children and parents were compensated for their participation to the amount of $200 US dollars per family (Garavan et al. 2018). Finally, interviews were proposed in English and in Spanish. ABCD received ethics approval from University of California San Diego (i.e., ABCD principal investigating site) and from review boards of other study sites.

### Study groups

In the present study we focused on data from mothers who were taking antidepressants before being aware of their pregnancy. For clarity, we refer to participants as “*biological mothers*”, although four participants identified as males. The inclusion criteria were, to be a biological mother and to have been using antidepressants before awareness of pregnancy. Based on information on antidepressant use after being aware of pregnancy (defined below), biological mothers were categorized as those who continued or discontinued antidepressant medication during pregnancy.

### Measurements

Participants were asked questions about their medication use before they were aware they were pregnant and during their pregnancy. The research staff systematically coded each medication reported by parents using their US brand name and code in the *RxNorm* system. We used *Rx* codes to get the corresponding Anatomical Therapeutic Chemical (ATC) classification codes and identified those corresponding to antidepressants (i.e., N06A in ATC classification). We established the presence of antidepressant use during, before and after awareness of pregnancy.

Based on the literature, we included relevant social determinants of health associated with antidepressant discontinuation in the general population (Olfson et al. 2006), including gender (male, female, male trans, female trans, non-binary, or other), first language, ethnicity, education, household income and living with a partner. We also included factors specific to perinatal health, namely age at childbirth, pregnancy duration and having a planned pregnancy. Of note, information on education, household income and having a partner covered the period 10 years after the pregnancy. It was used as a proxy as their sociodemographic status during pregnancy, though it might have changed within the past 10 years. We dichotomized each sociodemographic factor to describe whether participants were belonging to the majority or to minorities (e.g., female *versus* others, white *versus* non-white), except for age at childbirth and pregnancy duration. Thereby, gender was categorized as female vs. other. Maternal ethnicity was proxied by infant ethnicity and categorized as white vs. other. Education was recoded as having obtained a university diploma or not, literature having shown that antidepressant users in the US population often have a university level (Olfson et al. 2006). Household incomes were categorized as more than $75,000 US dollars per year or less. Of note, the median household incomes in 2005 to 2010 was of about $50,000 to $60,000, so that earning $75,000 or more could be considered as a high income. Finally, participants were asked if their pregnancy was planned. Smoking during pregnancy was used as a proxy for a lower perinatal health literacy given that the literature shows that smoking during pregnancy is associated with such a low perinatal health literacy (Smedberg et al. 2014) and that perceived risk of tobacco-based products is negatively associated with a higher perceived risk of antidepressants (Nordeng, Ystrom, and Einarson 2010; Petersen et al. 2015).

## Statistical analyses

Descriptive statistics included the mean and standard deviation or both frequency and proportion of each variable among the sample and between continuers and discontinuers. We performed simple logistic regression models to test respectively the crude associations (*odds ratios*) between continuation of antidepressant use and each factor. Then we tested the adjusted associations in a multiple logistic regression, and we measured the part of explained variance of antidepressant continuation explained by the model using Nagelkerke’s pseudo-*R*^*2*^ (Nagelkerke 1991). We conducted the analyses with a 5% threshold for significance and listwise deletion was performed in case of missing data.

We conducted data management and statistical analyses were using *R* statistical software (v.4.3.1), the packages *fmsb* (v.0.7.5) and *jtools* (v.2.2.2), and the package *rxnorm* developed by Nicholas T. Wiliams (version from May 22^nd^ 2023 https://github.com/nt-williams/rxnorm).

## Results

**Fig. 1:**
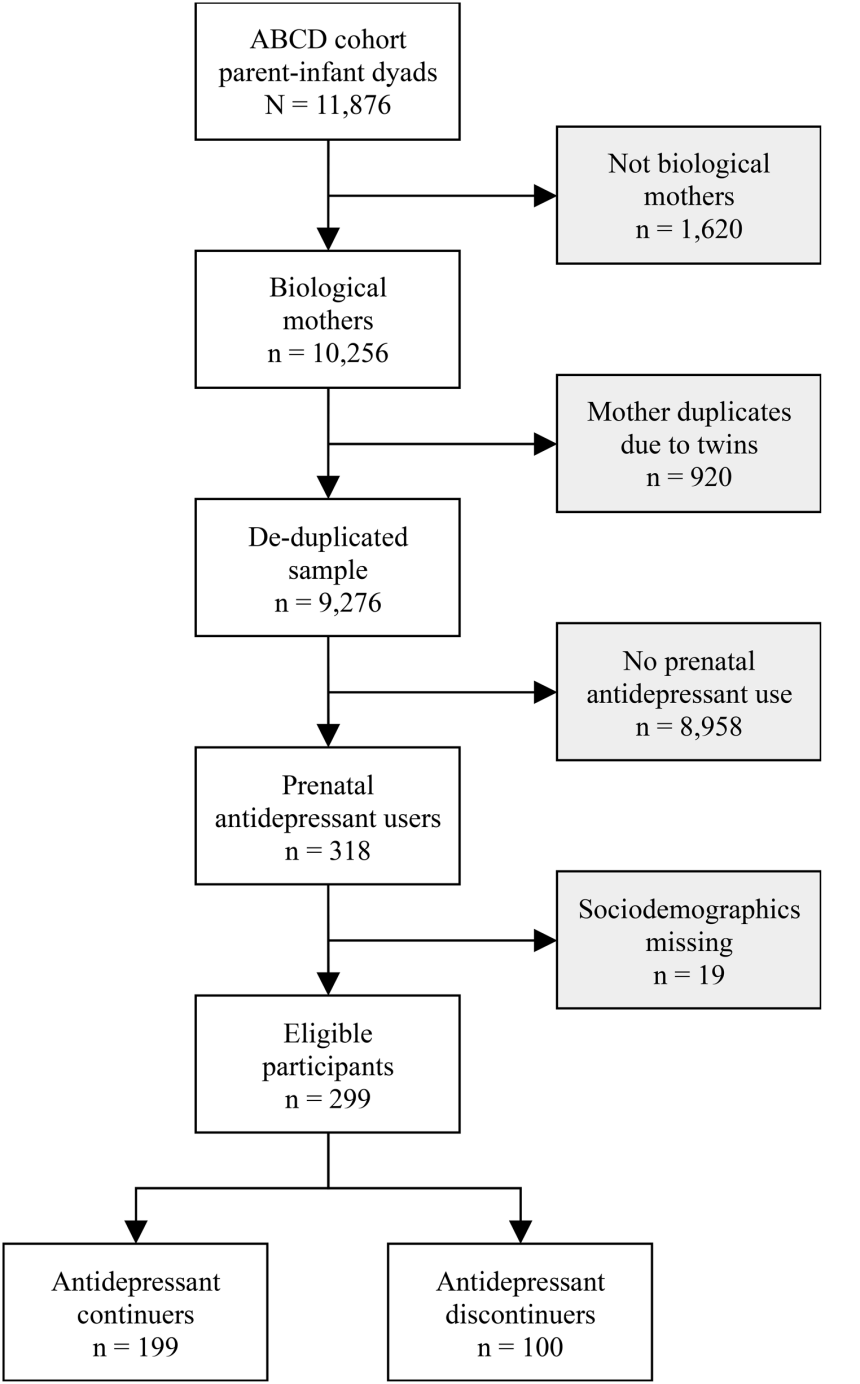
Flowchart of participant selection

As detailed in Fig. 1, among the 11,876 parent-infant dyads included in the ABCD cohort, 10,256 were biological mothers. Of them, 9,276 unique mothers were identified after de-duplicating mothers appearing multiple times because of twins or triplets. In total, 318 (3.42%) biological mothers were using antidepressant before being aware of their pregnancy, corresponding to the inclusion criterion. Of them, 19 had missing values regarding sociodemographic information and were excluded. The final sample consisted of 299 participants (94.0% of the identified antidepressant users). In total, 100 discontinued antidepressants and 199 continued antidepressants. Participants’ characteristics are reported in Table 1 for both groups. Mean age was 31.2 ± 5.5 years and mean duration of pregnancy was 272.6 ± 15.7 days. A total of 295 (98.7%) participants were women, 241 (80.6%) had a white ethnicity, and 197 (65.9%) had planned their pregnancy. At follow-up, 247 (82.6%) were living with a partner, 198 (65.2%) had no university diploma, and 208 (65.9%) had an inhouse income higher than $75,000 US dollars per year.

**Table 1:** Descriptive statistics of the participants

	Continuers (*n* = 199)	Discontinuers (*n* = 100)
*Age at childbirth [years]*	31.3 ± 5.4	31.1 ± 5.6
*Pregnancy duration [days]*	272.3 ± 16.0	273.3 ± 15.1
*Gender at follow-up*		
Female	195 (98.0%)	100 (100.0%)
Male	4 (2.0%)	-
*First language*		
English	197 (99.0%)	91 (91.0%)
Others^1^	2 (1.0%)	9 (9.0%)
French	1 (0.5%)	1 (1.0%)
Russian	-	1 (1.0%)
Spanish	1 (0.5%)	6 (6.0%)
Vietnamese	-	1 (1.0%)
*Ethnicity*		
White	165 (82.9%)	76 (76.0%)
Others^1^	34 (17.1%)	24 (24.0%)
Black	2 (1.0%)	2 (2.0%)
Hispanic	13 (6.5%)	13 (13.0%)
Asian	-	1 (1.0%)
Other ethnicities	19 (9.5%)	7 (7.0%)
*Education at follow-up*		
University diploma	65 (32.7%)	36 (36.0%)
No university diploma	134 (67.3%)	64 (64.0%)
*Household income at follow-up [US$ per year]*		
High (> 75,000)	140 (70.4%)	68 (68.0%)
Low to average^1^	59 (29.7%)	42 (42.0%)
Median (50,000 to 75,000)	26 (13.1%)	14 (14.0%)
Low (< 50,000)	33 (16.6%)	18 (18.0%)
*Living with partner at follow-up*		
Yes	173 (86.9%)	74 (74.0%)
No	26 (13.1%)	26 (26.0%)
*Planned pregnancy*		
Yes	140 (70.4%)	57 (57.0%)
No	58 (29.1%)	43 (43.0%)
Refused to say^2^	1 (0.5%)	-
*Smoking during pregnancy*		
Yes	27 (13.6%)	23 (23.0%)
No	171 (85.9%)	77 (77.0%)

^1^ The subcategories are detailed in this table, though they are regrouped for the later analyses

^2^ Recoded as “no”

Results from logistic regression analysis (Table 2) showed that four crude associations between factors and continuation of antidepressant medication were significant: English as the first language (*OR* = 9.74, *p* = .004), living with a partner (*OR* = 2.34, *p* = .006), planned pregnancy (*OR* = 0.56, *p* = .022) and smoking when pregnant (*OR* = 0.53, *p* = .043). Native English speakers and people living with a partner were more likely to continue antidepressant medication, whereas people who planned their pregnancy and were smoking while pregnant were more likely to discontinue. When testing the associations with these factors in a global model, only English as a first language had a significant association. The association was larger when adjusting for the other factors (*aOR* = 14.94, *p* = .015). The model resulted in a Nagelkerke pseudo*-R*^*2*^ of 11.3% (*p* = .005).

**Table 2:** Crude and adjusted associations between sociodemographic factors and antidepressant continuation

	*OR*	95% CI		*P*	*aOR*	95% CI		*p*
Age	1.01	0.96	1.05	0.718	1.01	0.96	1.06	0.819
Education	1.16	0.70	1.92	0.565	0.97	0.53	1.74	0.926
Ethnicity	1.53	0.84	2.75	0.155	1.05	0.52	2.04	0.886
First language (English)	9.74	2.45	64.74	0.004	14.94	2.40	291.45	0.015
Living with a partner	2.34	1.27	4.31	0.006	1.69	0.76	3.75	0.194
Household income	1.12	0.66	1.87	0.677	0.73	0.35	1.47	0.381
Planned pregnancy	0.56	0.34	0.92	0.022	0.61	0.35	1.06	0.079
Pregnancy duration	0.99	0.98	1.01	0.424	1.00	0.98	1.01	0.738
Smoking during pregnancy	0.53	0.28	0.99	0.043	0.59	0.30	1.19	0.136

*OR*: odds ratio; *aOR*: adjusted odds ratio; 95% CI: confidence interval

## Discussion

Pregnancy is a decisive moment in trajectories of discontinuation of medical treatments, but little research has been done to identify the factors associated with the continuation of medication in this specific period. We tested the associations between sociodemographic factors and the decision to continue antidepressant medication when becoming aware of pregnancy.

### Main findings

First, discontinuers accounted for about one third of women using antidepressants at pregnancy start. The proportion of women discontinuing antidepressants was 20–30% lower than in literature based on real-world data. However, literature so far comes from European countries that have achieved universal health coverage such as Denmark (Trinh et al. 2023), France (Cabaillot et al. 2021), Italy (Lupattelli et al. 2023) or Norway (Trinh et al. 2023). In the US context in the second half of the 2000s, it is likely that a large proportion of mental health care was forgone, which would explain the proportion of discontinuers found.

We found that English as the first language, in a country where English is the national language, was significantly associated with continuing antidepressants in a multiple logistic regression model. The odds ratio of 14.94 measured represents a very large association according to Chen and colleagues (2010). The model resulted in a determination coefficient of 11.3%, which indicates that the influence of sociodemographic factors on continuation of antidepressants is far from negligible in the US context.

In the USA, being a native English speaker seems to largely impact the decision to continue antidepressant medication during pregnancy. We discuss three possible explanations for this. Firstly, English speaking background is likely to play a role in the access to up-to-date information about health in a country where English is the first language. Accordingly, people from non-English speaking backgrounds may have less access to health information in their first language. They may also be less likely to look for information because they believe that information or counselling will not be available in their first language. Efforts to make information available in Spanish would probably reduce the difference, Spanish speakers accounting for about 13% of the US population (Census Bureau 2022). Second, being not a native English speaker also reflects family history of immigration and specific attitude toward health care between native English speakers and other people (who could be non-native English speakers or not speaking English at all). This also includes attitudes toward medication in pregnancy and toward mental health that may influence their decision, even in presence of up-to-date information. According to this second possible explanation, first language represents a correlate of confounding factors, not only as a causal factor. Third, differences in first language may reflect economic differences. Though controlled for, household income was measured at follow-up and income may not fully reflect wealth at baseline. For instance, it has been shown among ABCD participants that Spanish-speaking families experienced more financial concerns with the Covid-19 pandemic (Yip et al. 2022). Most likely, the three explanations apply together.

We also found non-significant associations where significant results were expected. This includes the negative association between having a planned pregnancy and continuing antidepressants. Though a large proportion of women discontinue antidepressants when aware of their pregnancy (Trinh et al. 2023; Trinh, Semark, Trinh et al. 2023a, b), the association might be not significant in this study when adjusting the model due to a lack of statistical power. The association between smoking and continuing antidepressants, though not significant, was negative in the adjusted model, which suggests that people continuing smoking also were likely to discontinue antidepressants. Though living with a partner and household income might have resulted in significant associations with a bigger sample, ethnicity and education seemed not to be associated with continuing or discontinuing antidepressants. It is likely that the underlying factors are associated with first language, education and ethnicity, which explains why education and ethnicity seem to have neither significant nor meaningful association with antidepressant continuation when taking first language into account.

Of note, other authors reported that pregnant women who start taking antidepressants in the USA (where health insurance is not mandatory) more frequently have an average-to-high household income, a slightly higher educational level and are older (Moreau et al. 2023), whereas major depression is more prevalent among people with a lower household income (Sareen et al. 2011). Obstacles to access antidepressants are likely to be smaller in countries with public or mandatory health insurance. Pregnant women with lower incomes or education levels are thus most likely to forgo mental health care (Bremer 2014), which is another major public health concern.

### Clinical implications

Though the underlying reasons might be multiple, the fact that native English speakers in this US based study were more likely to continue using antidepressants during pregnancy is a useful insight. Prescribers and pharmacists should be aware that people not speaking the native language of a country (or speaking it only a little) may discontinue their antidepressant medication during pregnancy. Native Spanish speakers might be targeted first given their proportion in the US population. Targeted communication to mental health professionals may also be useful to ensure that accurate harm information about antidepressant use is provided to all pregnant women. Importantly, our study excluded women who had come to the end of their antidepressant treatment and subsequently planned for their pregnancy. Thus, we hypothesize that a high proportion of treatment discontinuers in our study still needed the treatment. The consequences of discontinuing an antidepressant when medication is still required include a higher risk of relapse (Liu et al. 2022; Trinh et al. 2023a). Untreated affective disorders occurring during or before pregnancy have in common the risks of aggravation and chronicization (Fisher et al. 2019), and discontinuation of a needed treatment might be associated with similar risks. Moreover, the presence of affective disorders in mothers can also impact their child’s development with social interactions, impulsivity and conduct disorders (Stein et al. 2014).

### Limitations

There are some limitations related to the main study design. The first limitation is the retrospective data collection of maternal information. Regarding representativeness, ABCD consists of adolescents and their parents, which implies that mothers who had a miscarriage were not included. Antidepressant users might be underestimated because people suffering from chronic depression are less likely to consent to participate in observational studies (Dupuis et al. 2019).Interviews in Spanish were possible for Spanish-speaking mothers, which is a strength, but other foreign languages might be underrepresented.

In addition retrospective studies are exposed to recall bias (Dupuis et al. 2023), but previous literature showed that recall is accurate regarding antidepressant use in pregnancy (Newport et al. 2008). Even when people do not remember the exact brand name, they are able to state that they used antidepressants or psychotropic medication (van Gelder et al. 2013). If present in this study, recall bias most likely impacted remembering antidepressant use during pregnancy in general (that is to say *before and after* awareness of pregnancy). In other words, recall bias most likely caused non-inclusion of some antidepressant users given our inclusion criteria (i.e., reporting antidepressant use before being aware of pregnancy).

Study limitations also regard measurements. Mental health status during pregnancy would have been an important factor. As discussed, household income and living with a partner a decade later are not excellent proxies for the same outcomes during pregnancy. The non-significant association of marital status with antidepressant discontinuation may mostly be explained by changes in marital status over time. Cohabitation with a partner has an average duration of 7-to-8 year in the USA and has a large impact on household income. This makes household income likely to change more than individual income itself (Tach and Eads 2015).

### Recommendations for future research

Our findings are related to the specific context of the US population in the 2000s. The situation has most probably changed since the Covid-19 pandemic. Furthermore, the study is based on secondary data collected retrospectively, which is not optimal. Though the findings result from multi-stage probability sampling, the sample remains small compared to the number of women exposed to prenatal antidepressants. Our findings merit being reproduced using other methods, in particular a prospective design. Subject to the availability of sociodemographic characteristics, real-world data from health insurances would represent an opportunity to confirm the present findings at a national level and to investigate the influence of other social determinants with sufficient statistical power.

## Conclusion

Despite its limitations, the present study identified that speaking the native language of the country seems an important factor associated with continuing antidepressant medication during pregnancy. Based on this finding, information to people who are not native English speakers could be improved to reduce inequities among pregnant patients in the USA.
